# Global research landscape of antiangiogenic therapy for colorectal cancer: a bibliometric analysis of mechanistic insights and clinical advancements

**DOI:** 10.3389/fonc.2025.1591059

**Published:** 2025-07-24

**Authors:** Ke-Qiang Hou, Jia-Ming Wu, Jie Chen

**Affiliations:** ^1^ Shanghai Institute of Materia Medica, Chinese Academy of Sciences, Shanghai, China; ^2^ Department of Gastrointestinal Surgery, The First Affiliated Hospital of Jiaxing University, Jiaxing, Zhejiang, China; ^3^ Department of Central Laboratory, The First Affiliated Hospital of Jiaxing University, Jiaxing, Zhejiang, China

**Keywords:** colorectal cancer, antiangiogenesis therapy, basic mechanism, clinical treatment, bibliometric analysis, VOSviewer, CiteSpace

## Abstract

**Background:**

Colorectal cancer (CRC) is a major global health issue, with over 1.9 million diagnoses yearly and low survival rates in advanced stages. Antiangiogenic therapies (AAT) targeting VEGF and VEGFR have improved outcomes, but resistance mechanisms limit their effectiveness. This study uses bibliometric analysis to link mechanistic insights, such as VEGF splicing variants, with clinical developments, identify global collaboration trends, and propose strategies to reduce resistance and toxicity in treatments.

**Methods:**

This study were used to search the Web of Science databases Core Collection. Studies published in English from 1996 to 2024 were included for analysis. VOSviewer 1.6.20, CiteSpace 6.4.R1, and R 4.4.1 were employed for bibliometric analysis and visualization.

**Results:**

This bibliometric analysis of 976 publications from 1996 to 2024 shows a 13.65% annual growth rate in CRC antiangiogenic research. China leads with 20.5% of publications, followed by the USA at 15.7% and Japan at 13.1%. Key institutions include Assistance Publique Hôpitaux de Paris, and notable journals are BMC Cancer and Clinical Colorectal Cancer. Keyword evolution reflects a shift from angiogenesis mechanisms to clinical validation of treatments like FOLFIRI with bevacizumab, with a current focus on tumor microenvironment reprogramming and precision survival analytics (2020-2024, burst intensity 6.66). Key milestones include Phase III trials like AVF2107g and ctDNA-guided strategies, along with emerging dual-target inhibitors.

**Conclusion:**

This bibliometric analysis reveals a shift from VEGF studies to precision strategies targeting tumor microenvironments, influenced by trials like TRIBE and PARADIGM. Future efforts should focus on multi-omics integration and innovative delivery systems like circadian-targeted nanoparticles for personalized CRC care.

## 1 Introduction

Colorectal cancer (CRC) represents a significant global health challenge, with annual incidences exceeding 1.9 million new cases and more than 900,000 fatalities. This concerning statistic underscores the urgent necessity for the development of innovative therapeutic strategies (1, 2). Although conventional treatments, including surgical resection, chemotherapy, and radiotherapy, enhance outcomes in early-stage disease, the five-year survival rate for metastatic colorectal cancer (mCRC) remains below 15%. This scenario accentuates the imperative to explore novel treatment modalities (3, 4). The advent of antiangiogenic therapy (AAT), inspired by Folkman’s hypothesis postulating that tumors rely on angiogenesis (5), has revolutionized the management of CRC by targeting the vascular endothelial growth factor (VEGF) and its receptor (VEGFR) axis, which are pivotal for tumor angiogenesis and progression (6–8). Bevacizumab, the inaugural antiangiogenic agent approved by the FDA, has augmented the median overall survival for mCRC from 15.6 months with chemotherapy alone to 20.3 months (9–11). Recent advancements in molecular profiling have elucidated that splice variants of VEGF-A165b and the conformational dynamics of VEGFR2/Neuropilin-1 considerably influence therapeutic variability, thereby paving the way for precision medicine (12–14). Nevertheless, the translation of these findings into clinical practice is fraught with challenges: approximately 30% of patients develop resistance via cancer-associated fibroblast-derived IL-8/PI3K/Akt/mTOR signaling pathways, and the transient nature of vascular normalization (2-4 weeks post-treatment) necessitates the optimization of chemotherapy scheduling (15–17).

To mitigate this resistance, combinatorial treatment strategies are gaining prominence. The FRESCO-2 trial demonstrated that dual inhibition of VEGFR2 and TIE2 through regorafenib, in conjunction with anti-EGFR therapy, extends median survival to 12 months in RAS wild-type mCRC (18, 19). Innovations in liquid biopsy methodologies, as highlighted by the CIRCULATE-PRO study, now incorporate circulating tumor DNA (ctDNA)-based ANGPT2/VEGFA biomarkers (AUC=0.82) into National Comprehensive Cancer Network (NCCN) guidelines, thereby facilitating personalized aflibercept therapy (20, 21). Furthermore, advancements in nanotechnology are addressing drug delivery challenges; pH-sensitive carriers for VEGFR inhibitors can enhance tumor drug concentration fivefold while concurrently reducing cardiotoxicity by 40% (22).

Despite these advancements, research pertaining to antiangiogenic therapies remains fragmented, with significant gaps existing between mechanistic discoveries and clinical applications. Previous bibliometric analyses of CRC therapies have not systematically examined specific trends, collaborative efforts, and the evolution of knowledge within antiangiogenic research (23, 24). Employing mathematical and statistical methodologies, bibliometric analysis yields insights into publication trends, key research areas, co-authorship dynamics, keyword frequency, and citations over time, thereby providing a comprehensive overview of the academic landscape surrounding AAT in CRC (25–27). It elucidates interdisciplinary connections (such as those between cancer-associated fibroblast biology and drug resistance trials), identifies underexplored targets (notably TIE2 signaling), and anticipates emerging research domains, including microenvironment reprogramming and dual-target inhibitors (28, 29). The objective of this study is to conduct a comprehensive bibliometric analysis of AAT in CRC, focusing on the following aims: 1)To delineate the global research landscape, encompassing contributions from various countries and institutions as well as collaborative networks. 2)To trace the evolution of mechanistic insights (e.g., VEGF splicing variants) to clinical milestones (e.g., biomarker-guided trials). 3)To predict future trends through keyword burst detection, thereby informing strategies to address resistance and toxicity.

## 2 Methods

### 2.1 Literature search and data identification

A comprehensive literature search was conducted utilizing the Web of Science Core Collection (WoSCC) database, which is esteemed for its rigorously vetted journals spanning diverse academic disciplines. This search, performed on February 4, 2025, aimed to identify studies published between January 1, 1996, and February 4, 2025. The search strategy employed for investigating the fundamental mechanisms included the following formulation: TS=(((“antiangiogen*” OR “anti-angiogen*” OR “angiogenesis inhibitor*” OR “VEGF inhibitor*” OR “VEGFR antagonist*” OR “vascular endothelial growth factor target*” OR “bevacizumab” OR “sunitinib” OR “sorafenib” OR “aflibercept” OR “ramucirumab”) NEAR/3 (therap* OR treat* OR agent* OR target* OR inhibit* OR block*)) AND ((“colorectal neoplas*” OR “colorectal cancer*” OR “colorectal carcinom*” OR “CRC” OR “bowel cancer*” OR “colorectal adenocarcinoma*”) NOT (“mouse” OR “rat” OR “murine”)) AND (“mechanism of action” OR “signaling pathway” OR “molecular mechanism” OR “VEGF/VEGFR axis” OR “tumor microenvironment” OR “hypoxia” OR “drug resistance” OR “metastasis” OR “epigenetic regulation”)) NOT TS=(“plant” OR “insect” OR “clinical trial” OR “case report” OR “review”). The search strategy for clinical treatments was structured as follows:TS=(((“antiangiogen*” OR “anti-angiogen*” OR “angiogenesis inhibitor*” OR “VEGF inhibitor*” OR “VEGFR antagonist*” OR “vascular endothelial growth factor target*” OR “bevacizumab” OR “aflibercept” OR “ramucirumab” OR “regorafenib” OR “sunitinib” OR “sorafenib” OR “pazopanib”) NEAR/3 (therap* OR treat* OR agent* OR target* OR combin* OR synerg*)) AND ((“colorectal cancer*” OR “colorectal carcinoma*” OR “colorectal neoplasm*” OR “colorectal tumor*” OR “CRC” OR “bowel cancer*” OR “colorectal adenocarcinoma*” OR “metastatic colorectal cancer*” OR “mCRC”) NEAR/3 (“clinical trial*” OR “phase I” OR “phase II” OR “phase III” OR “randomized controlled trial*” OR “RCT” OR “first-line” OR “second-line” OR “adjuvant” OR “palliative” OR “overall survival” OR “OS” OR “progression-free survival” OR “PFS”))) NOT TS=(“plant” OR “insect” OR “mouse” OR “rat” OR “murine” OR “animal model*” OR “*in vitro*” OR “*in vivo*”). The final logical conjunction utilized “OR” to ensure both comprehensiveness and precision. To uphold data consistency and accuracy, the search was restricted to articles published in English, encompassing only original research studies.

### 2.2 Data analysis and visualization

In this study, three bibliometric tools were employed for data analysis and visualization: VOSviewer version 1.6.20, CiteSpace version 6.4.R1, and the R-based package “bibliometrix.” Each tool fulfilled specific analytical functions, enabling a comprehensive exploration of the AAT in CRC research. VOSviewer facilitated the generation of visual representations of collaborative networks, which encompassed author and institutional collaborations, co-authorship networks, citation patterns, keyword co-occurrence, and co-citation clusters (30). Within these visualizations, the size of the nodes corresponded to the number of publications or citations, node color denoted cluster groupings, and link thickness represented the strength of relationships. This methodological framework aided in the identification of influential entities and emerging research trends within the academic sphere. CiteSpace was utilized to detect research trends and emerging hotspots through keyword burst analysis and betweenness centrality (31). The analysis encompassed the period from 1996 to 2024, focusing primarily on keywords as the node type. Network pruning techniques, including pathfinder and clip merge, were applied to refine the visualizations and enhance their interpretability, thereby enabling the identification of significant shifts in research focus and highlighting nascent areas of interest related to AAT in CRC research. The R-based package “bibliometrix” was employed for a comprehensive evaluation of research output, global distributions, and the performance metrics of authors and journals. These metrics yielded valuable insights into the academic influence and prestige of journals within the field (32). By integrating these analytical methodologies, this study provides a systematic overview of AAT in the CRC research landscape, emphasizing key contributors, prominent institutions, and evolving research trajectories. The findings impart valuable guidance for future investigations and foster collaborative opportunities aimed at enhancing the understanding and treatment of CRC.

## 3 Results

### 3.1 Overview of publications and trends

**Figure 1:**
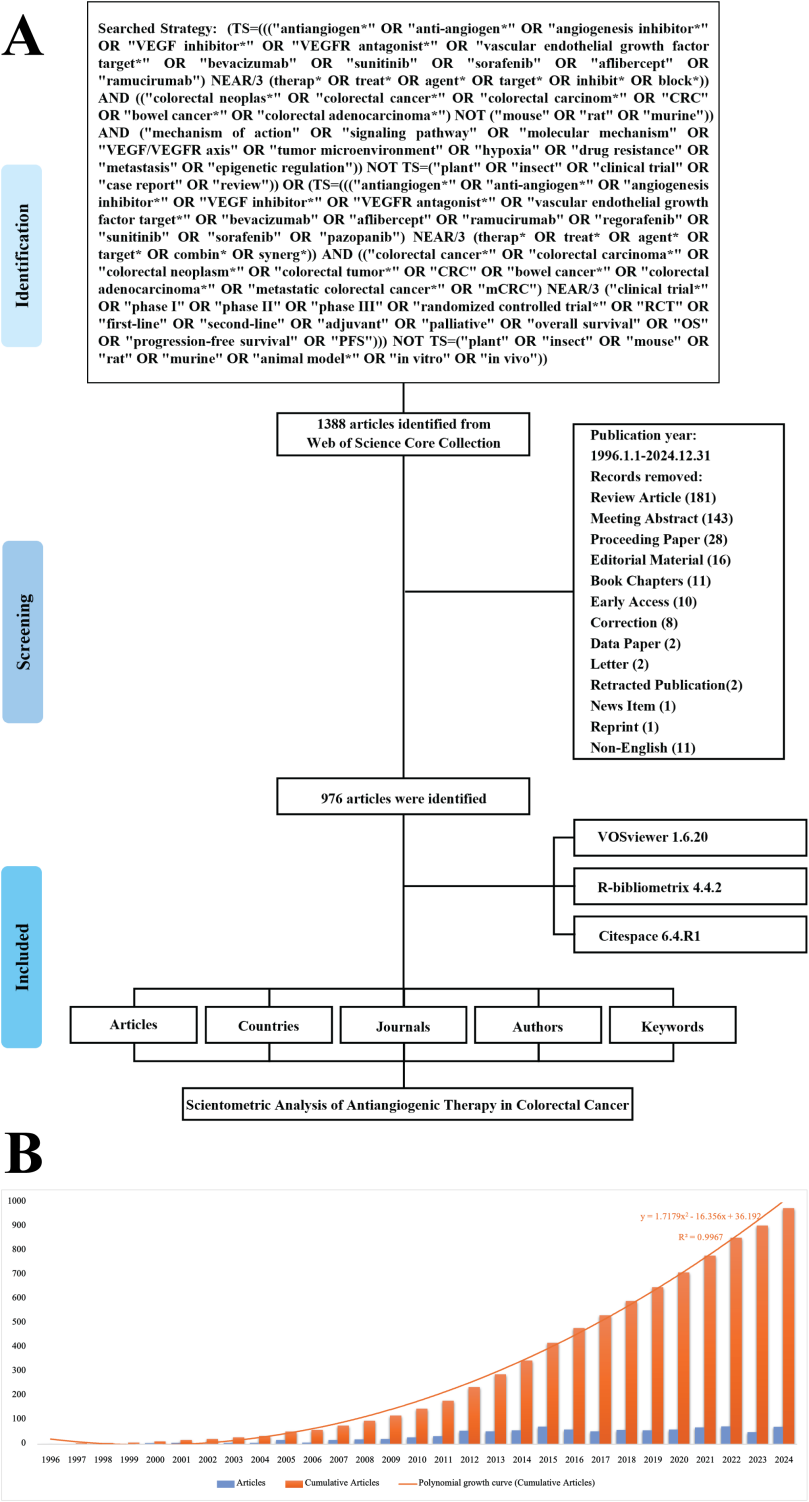
Overview of publication trends. **(A)** Literature screening process. **(B)** Annual publication volume and growth trend.

The comprehensive selection process is delineated **Figure 1A**. Between 1996 and 2024, a total of 976 publications were released, reflecting a sustained interest in this research domain, characterized by an annual growth rate of 13.65%. These publications involved 7,537 authors, of whom 22.34% participated in international co-authorship, underscoring significant global collaboration. On average, each document contained 10.4 co-authors, and a total of 21,423 references were cited, supported by 1,570 distinct keywords, thereby illustrating the depth and diversity of the research. As depicted in **Figure 1B**, the evident growth trend in publication numbers closely follows the function y = 1.7179x^2^ - 16.356x + 36.192 (R² = 0.9967). The highest number of publications was recorded in 2022, reaching 74, followed by a slight decline to 50 in 2023 and an increase to 72 in 2024. The average number of citations per document is 38.7, indicating the enduring impact of these works. Furthermore, an average document age of 8.84 years emphasizes the ongoing relevance and advancement of the field within the research community.

### 3.2 Analysis of countries

**Figure 2:**
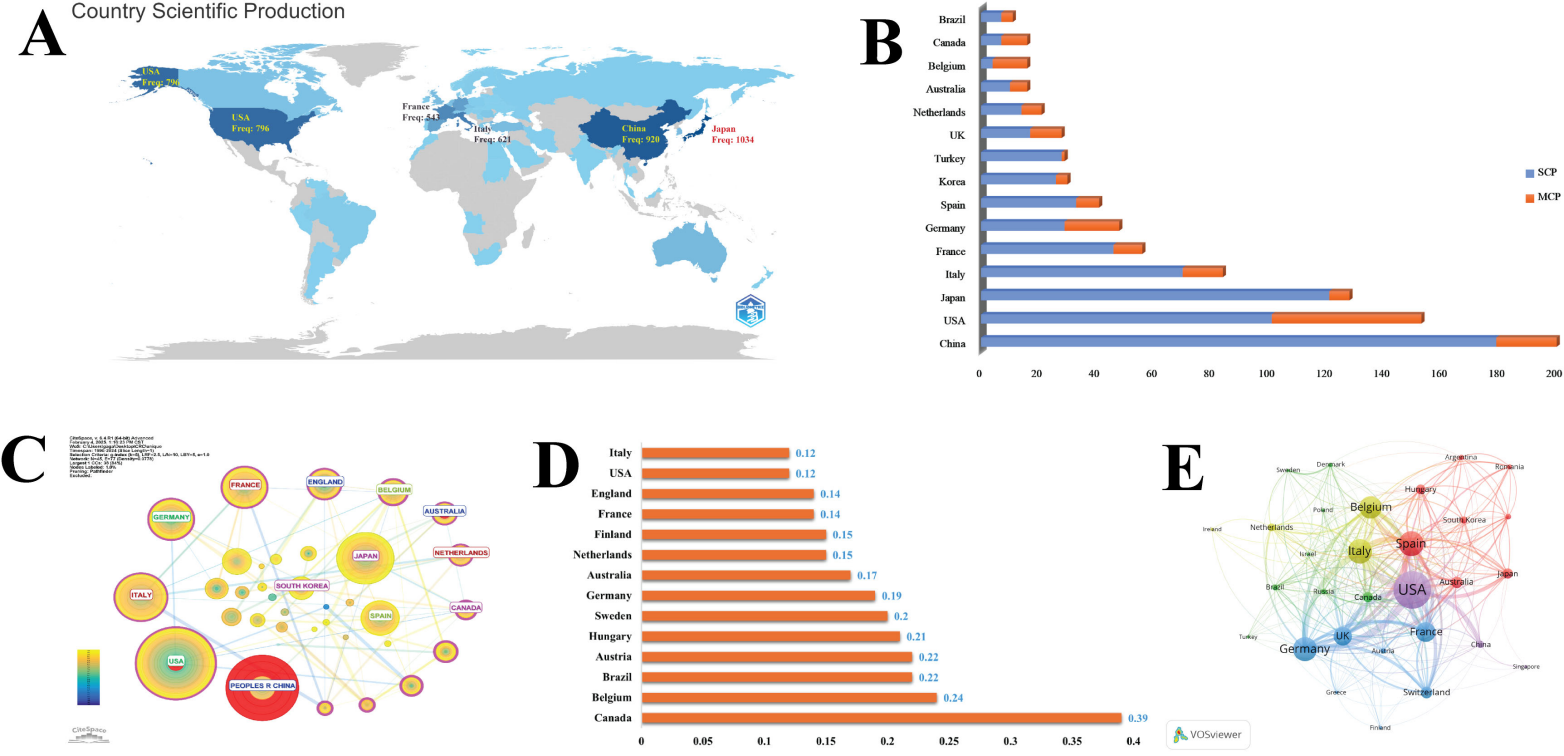
Country-level contributions and collaboration networks. **(A)** Geographical distribution of publications. **(B)** Global publication share. **(C, D)** Betweenness centrality of countries and rankings (>0.1) (citespace visualization) **(E)** International collaboration network (VOSviewer visualization).

**Figure 2A** depicts the geographical distribution of nations that have published at least one work in this domain over time. A blue intensity scale indicates the volume of records, ranging from 1 to 1,034, with darker shades representing a higher number of publications. Importantly, the nationality of all authors contributing to this compilation is considered. Japan ranks first with the highest number of publications (n = 1,034), followed by China (n = 920) and the USA (n = 796). The global distribution of publications reveals significant contributions from various countries. China leads with 200 articles (20.50%), followed by the USA with 153 articles (15.70%) and Japan with 128 articles (13.10%) (**Figure 2B**). Despite its substantial output, China exhibits a relatively low multiple-country publication (MCP) ratio of 0.105, suggesting limited international collaboration. In contrast, Canada demonstrates a robust MCP ratio of 0.563. Other nations, such as Germany (MCP ratio: 0.396) and the UK (0.393), also display significant collaborative efforts, underscoring the increasing importance of cross-border research partnerships in this field. As illustrated in **Figures 2C, D**, fourteen countries exhibit high betweenness centrality (> 0.1), ranked in descending order as Canada, Belgium, Brazil, Austria, and Hungary. Furthermore, VOSviewer has facilitated the visualization of international collaborations among countries, indicating that the USA, Italy, and Spain constitute the most robust international collaboration network, as depicted in **Figure 2E**.

### 3.3 Analysis of institutions

**Figure 3:**
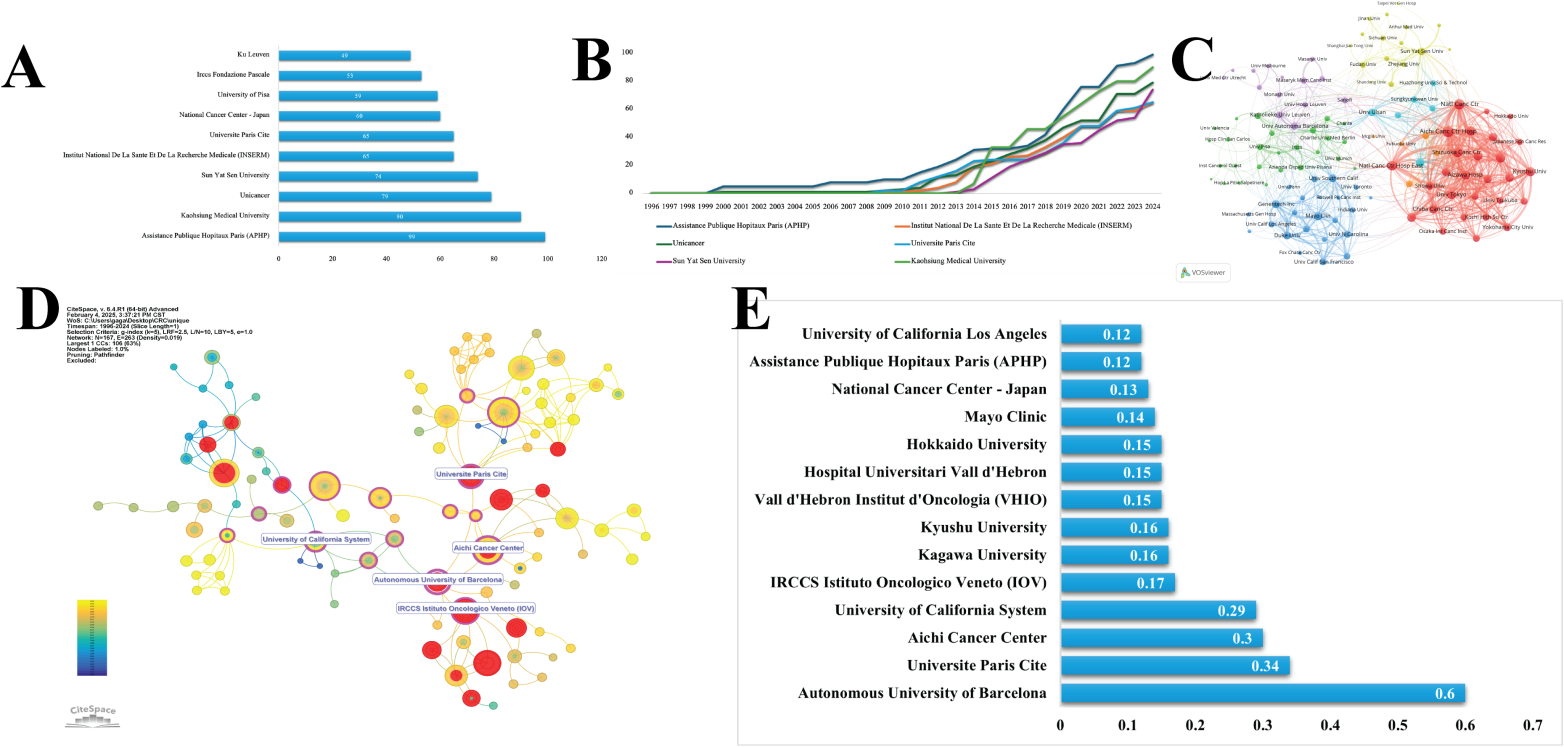
Institutional contributions and networks. **(A, B)** Institutional output and emerging trends. **(C)** Institutional collaboration network. **(D, E)** High betweenness centrality institutions and rankings (>0.1).

Institutional analysis indicates that Assistance Publique Hôpitaux de Paris (APHP) is the leading contributor, with a total of 99 articles, followed by Kaohsiung Medical University and Unicancer. Additionally, the analysis reveals emerging trends, as illustrated in **Figures 3A, B**. These institutions are central to the research network, forming dense collaborative clusters with both national and international partners. This pattern underscores their significant role in advancing research in this area and promoting global academic cooperation (**Figure 3C**). As illustrated in **Figures 3D, E**, fourteen institutions exhibit high betweenness centrality (> 0.1), ranked in descending order as Autonomous University of Barcelona, Universite Paris Cite, Aichi Cancer Center, University of California System, and IRCCS Istituto Oncologico Veneto.

### 3.4 Analysis of journals

**Figure 4:**
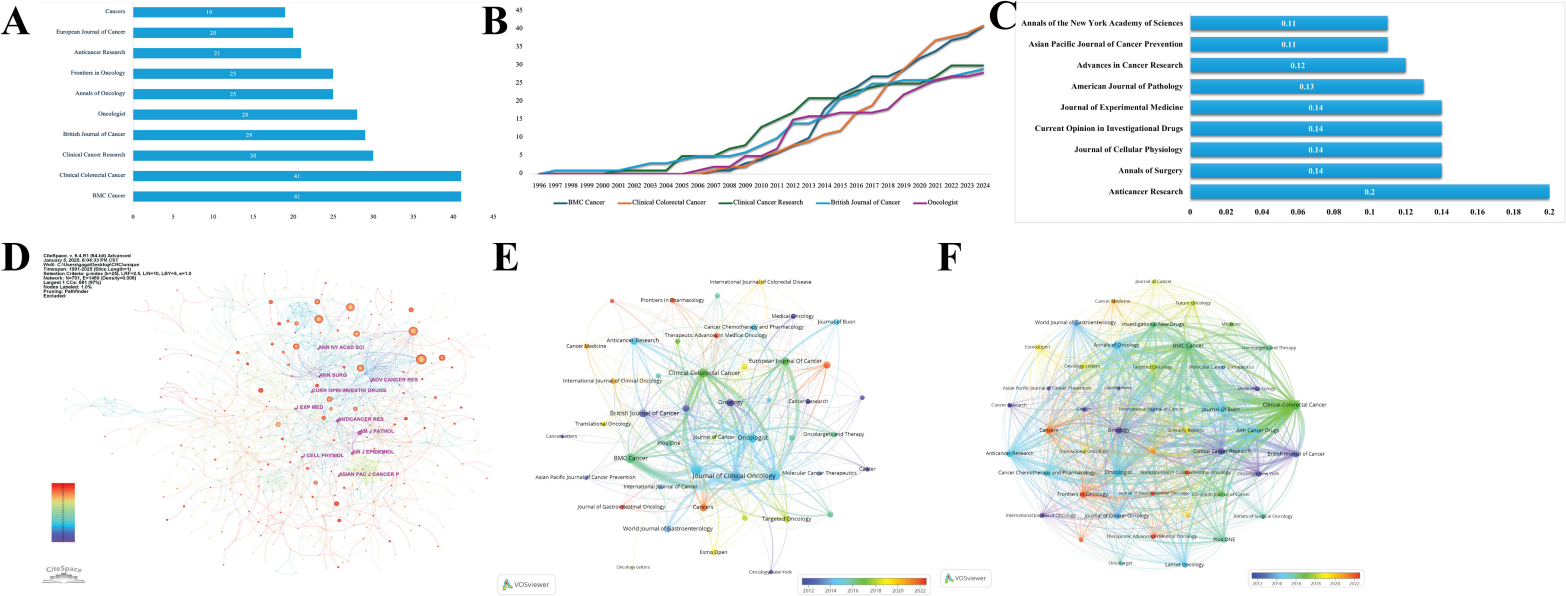
Journal influence analysis. **(A)** Journal publication distribution. **(B)** Emerging journal trends. **(C, D)** High betweenness centrality journals (>0.1) **(E, F)** Journal co-occurrence networks and bibliographic coupling.

The research articles examined in this study are published across 304 distinct journals. The most prominent venues include *BMC Cancer* and *Clinical Colorectal Cancer*, which collectively account for 41 articles. These are followed by *Clinical Cancer Research* with 30 articles and the *British Journal of Cancer*, as illustrated in **Figure 4A**. The analysis further reveals emerging trends, as depicted in **Figures 4B**, underscoring the emphasis of these journals on oncology. **Figures 4C, D** demonstrate that nine journals exhibit high betweenness centrality (> 0.1), ranked in descending order as follows: *Anticancer Research*, *Annals of Surgery*, *Journal of Cellular Physiology*, *Current Opinion in Investigational Drugs*, and *Journal of Experimental Medicine*. The co-occurrence network analysis identifies key journals, such as *BMC Cancer*, *Clinical Colorectal Cancer*, and the *Journal of Clinical Oncology*, as pivotal nodes, signifying their substantial interconnections within the discipline of Oncology. In the coupling networks, a total of 47 journals exhibit a minimum of five connections. Notably, the three journals with the highest total link strength in the co-occurrence networks are the *Clinical Colorectal Cancer* (15,812), *BMC Cancer* (15,808), and *Oncologist* (11,209). This distribution highlights the substantial contributions of these high-impact journals in shaping the research landscape (**Figures 4E, F**).

### 3.5 Analysis of authors

**Figure 5:**
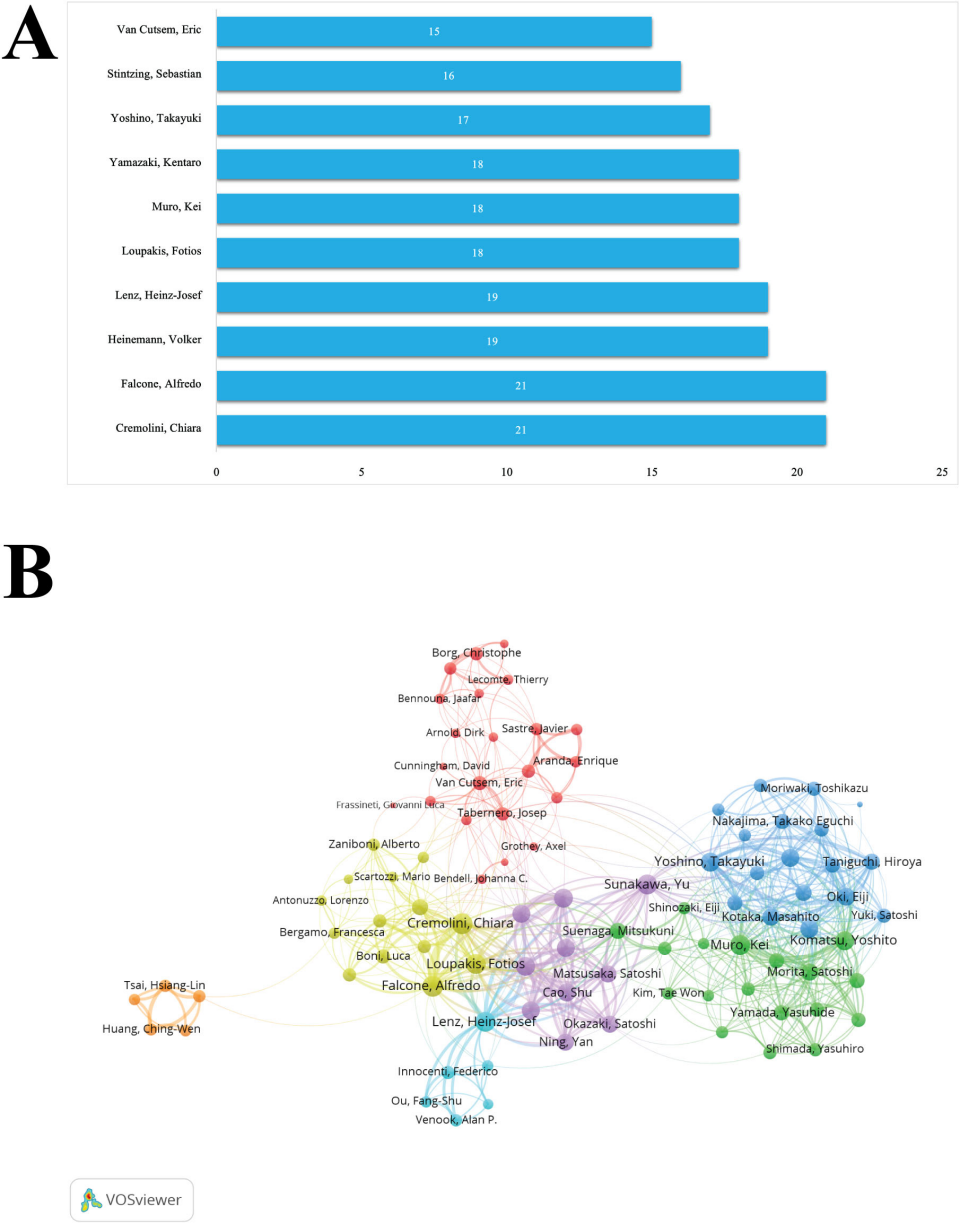
Author contributions and collaboration. **(A)** Top authors by publication volume. **(B)** Author collaboration network.

A total of 7,028 authors have contributed to this field. Among them, Cremolini, Chiara and Falcone, Alfredo ranks first with 21 publications (**Figure 5A**). In terms of collaboration and network influence (**Figure 5B**), Cremolini, Chiara also leads with the highest collaboration frequency. From second to fifth place, researchers including Falcone, Alfredo, Lenz, Heinz-Josef, Muro, Kei, and Loupakis, Fotios, show significant collaborative networks and are closely linked in terms of academic partnerships. However, when considering all factors, Cremolini, Chiara emerges as the most influential researcher in the field, demonstrating strong academic impact and leadership within these networks.

### 3.6 Analysis of keywords

**Figure 6:**
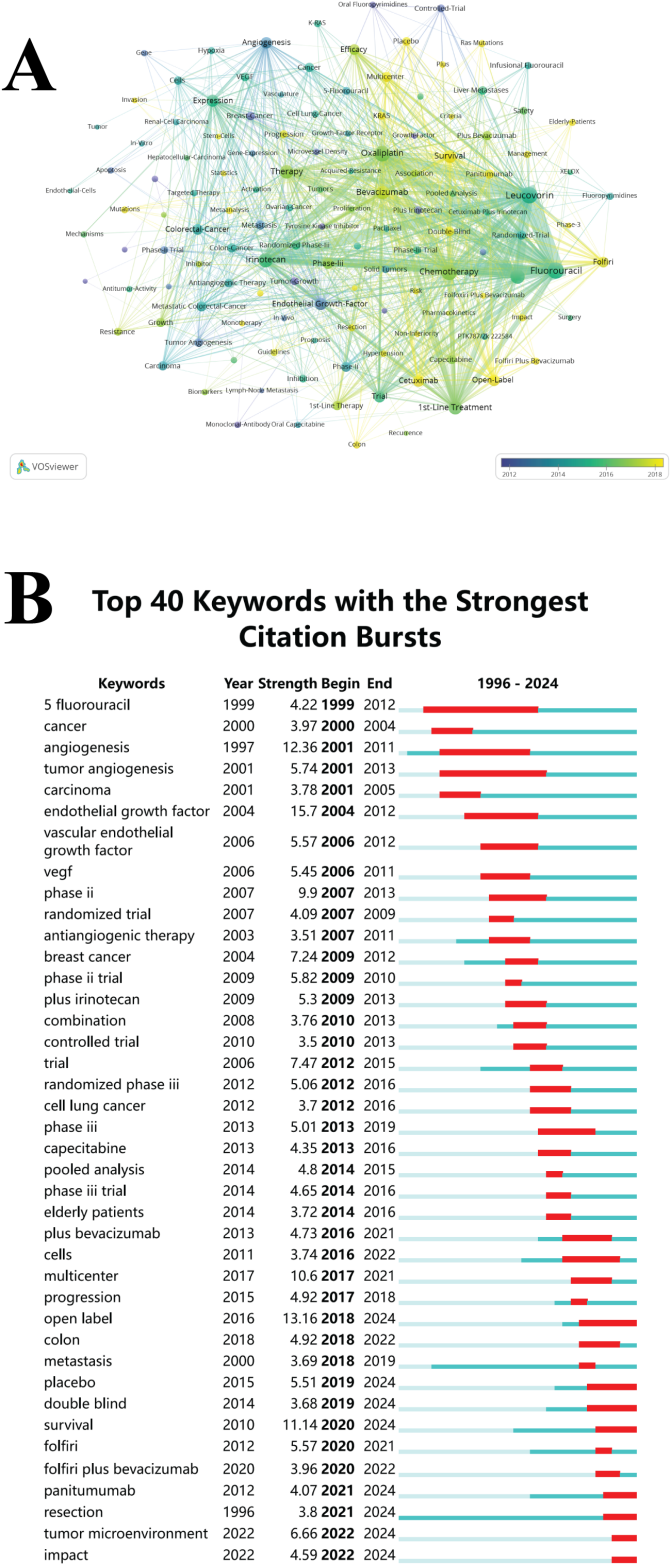
Keyword evolution and hotspots. **(A)** Keyword co-occurrence network (1996–2024). **(B)** Keyword burst analysis (citespace).

The keyword analysis reveals that the research domain primarily centers on spinal AAT in CRC, as depicted in **Figure 6A**. Keywords from the years 2010, 2014, and 2018 are color-coded in purple, green, and yellow, respectively, with disease-related terms omitted. In 2012, research hotspots emerged around terms such as “Oral Fluoropyrimidines,” “Monoclonal-Antibody,” and “Controlled-Trial,” underscoring an increasing focus on optimizing chemotherapy delivery systems—particularly oral formulations—and molecular-targeted therapies, including anti-EGFR and VEGF agents, for colorectal cancer treatment. These trends indicate a shift in the translational medicine paradigm towards validating combination regimens through large-scale randomized trials, especially for evaluating progression-free survival endpoints and safety profiles. The emphasis on controlled trials signifies a commitment to establishing evidence-based standards for targeted drug dosing and sequencing strategies in metastatic contexts.

By 2016, keywords such as “Leucovorin-modulated Fluorouracil dosing,” “Trinotecan chronotherapy,” and “oxaliplatin neurotoxicity mitigation” garnered significant attention, reflecting a transition toward precision scheduling of chemotherapy agents (e.g., circadian rhythm-guided Trinotecan infusion), prevention of treatment-induced neuronal apoptosis (e.g., through Bcl-2 upregulation), and the reconstruction of chemotherapy-damaged enteric neural networks. After 2018, the focus further shifted toward “Survival,” “Elderly-Patients,” and “Cetuximab,” indicating an increasing emphasis on monoclonal antibodies and prognostic management in this field.

CiteSpace identified 40 keywords exhibiting citation bursts, which illustrate the evolution of research hotspots with varying intensities (**Figure 6B**). Since the early 2000s, terms related to angiogenesis mechanisms, such as “angiogenesis” (burst intensity 12.36) and “vascular endothelial growth factor” (15.7), have been prominent, suggesting that early foundational research concentrated on tumor blood supply mechanisms. As clinical translation advanced between 2006 and 2013, keywords such as “antiangiogenic therapy” (3.51) and optimized chemotherapy regimens, including “5-fluorouracil” (4.22) and “capecitabine” (4.35), exhibited coordinated bursts, reflecting the concurrent application of anti-angiogenic agents, such as bevacizumab, alongside fluorouracil-based chemotherapy.

Around 2015, more comprehensive research was evidenced by bursts in keywords such as “randomized phase III” (5.06) and “plus bevacizumab” (4.73), marking a period of systematic validation in key Phase III clinical trials, including the AVF2107g and TRIBE studies. In recent years (2020-2024), “tumor microenvironment” (6.66) and “survival” (sustained burst intensity 11.14) have emerged as the most dynamic research areas, indicating a strategic shift toward targeting the tumor microenvironment and conducting precise survival analyses. Notably, the ongoing focus on established regimens like “FOLFIRI plus bevacizumab” (3.96) emphasizes the foundational role of anti-angiogenic combination chemotherapy in metastatic colorectal cancer.

This evolutionary map delineates a three-stage transition in cancer treatment research: from early breakthroughs in fundamental angiogenesis theory (early 2000s) to clinical validation of combination treatment regimens (mid-2010s), and the exploration of microenvironment targeting and precision treatment strategies (2020s). The burst keywords at each stage align with landmark clinical trials, such as the ECOG 2100 study of bevacizumab (burst in 2006), the molecular typing exploration of the FIRE-3 study (2013), and the differential analysis of left and right-sided colon cancer in the PARADIGM trial (2022).

## 4 Discussion

The evolution of AAT in CRC research has transitioned from foundational mechanistic studies to investigations with a more clinical focus. This transformation is evidenced by a bibliometric analysis of 976 publications covering the period from 1996 to 2024. Initially, research predominantly centered on VEGF and its corresponding receptor signaling pathways; however, it has since broadened to encompass advanced topics such as tumor microenvironment modulation, biomarker-guided therapies, and combination strategies that integrate immunotherapy with nanotechnology-based drug delivery systems.

### 4.1 Comparison of anti-angiogenic agents

Bevacizumab is recognized as a fundamental component of therapy among anti-angiogenic agents (AAT agents), with numerous clinical trials substantiating its efficacy when administered in conjunction with standard chemotherapy regimens such as FOLFOX and FOLFIRI. For instance, the AVF2107g trial demonstrated that the addition of bevacizumab to irinotecan-based chemotherapy significantly enhances overall survival in patients diagnosed with metastatic CRC (9). Similarly, the ML18147 study indicated that the continuation of bevacizumab beyond the initial progression could further prolong survival (34). Conversely, regorafenib, a multikinase inhibitor that targets vascular endothelial growth factor receptor (VEGFR), TIE2, and other signaling pathways, has exhibited promise in late-line treatment settings. The CORRECT trial revealed that regorafenib improved overall survival in patients with previously treated metastatic CRC (18). Furthermore, aflibercept, another agent targeting VEGF, demonstrated enhanced survival outcomes when combined with FOLFIRI in the VELOUR trial (10). These agents exhibit differences not only in their molecular targets but also in their side effect profiles and optimal sequencing within treatment algorithms. For example, bevacizumab is associated with an increased risk of hypertension and proteinuria, while regorafenib has been linked to hand-foot skin reactions and fatigue. Understanding these distinctions is crucial for personalized treatment planning. To facilitate this understanding, we have provided a comparative overview of the major AAT agents in **Table 1**. This table summarizes the mechanisms of action, key clinical trials, indications, and adverse effects of the principal AAT agents utilized in CRC, thereby highlighting their distinct roles within the treatment landscape.

**Table 1:** Comparative analysis of principal antiangiogenic therapeutic agents in colorectal cancer.

Agent	Mechanism of Action	Key Clinical Trials	Primary Indications	Common Side Effects
Bevacizumab	VEGF-A antibody	AVF2107g, ML18147	First-line mCRC with chemotherapy	Hypertension, proteinuria, bleeding
Regorafenib	Multikinase inhibitor (VEGFR, TIE2, etc.)	CORRECT, CONCUR	Third-line mCRC	Hand-foot skin reaction, fatigue, hypertension
Aflibercept	VEGF-A, B, PLGF trap	VELOUR	Second-line mCRC with FOLFIRI	Hypertension, proteinuria, diarrhea
Ramucirumab	VEGFR-2 antibody	RAISE	Second-line mCRC with FOLFIRI	Hypertension, fatigue, diarrhea

### 4.2 Highly cited literatures

In 2003, Fairooz Kabbinavar et al. (33) conducted a pivotal Phase II randomized clinical trial to evaluate the efficacy of the anti-vascular endothelial growth factor monoclonal antibody bevacizumab in conjunction with the fluorouracil/leucovorin (FU/LV) regimen for patients diagnosed with metastatic colorectal cancer. The findings indicated that the cohort administered 5 mg/kg of bevacizumab exhibited a statistically significant extension in median progression-free survival compared to the chemotherapy-only group (9.0 months vs. 5.2 months, P = 0.005). Additionally, this group demonstrated an increased objective response rate (40% vs. 17%) and a median overall survival advantage of 21.5 months. Despite certain limitations, including a relatively small sample size (n = 104) and imbalanced baseline characteristics, this trial was the inaugural study to establish that anti-angiogenic therapy could enhance the efficacy of conventional chemotherapy. This discovery provided a foundation for subsequent Phase III clinical trials, such as the AVF2107g study. Notably, the enhanced efficacy observed in the low-dose group (5 mg/kg) suggested the existence of a potential dose-effect relationship, which is essential for optimizing the integration of targeted therapies with chemotherapy regimens. Consequently, bevacizumab became the first anti-angiogenic agent to receive approval for the treatment of colorectal cancer. Moreover, the study underscored safety concerns, including thromboembolic events, thereby highlighting the necessity for vigilant clinical monitoring.

In 2012, the Phase III clinical trial ML18147, conducted by Bennouna et al. (34), evaluated the efficacy of continuous bevacizumab treatment following the progression of first-line therapy for metastatic colorectal cancer (mCRC). The study enrolled 820 patients who were randomly assigned to receive either second-line chemotherapy combined with bevacizumab or chemotherapy alone. Results indicated a significant extension in median overall survival for the combination group (11.2 months vs. 9.8 months, HR = 0.81, p = 0.0062) and an improvement in progression-free survival (5.7 months vs. 4.1 months, p < 0.0001), but the incidences of bleeding (2% vs <1%) and thromboembolic events (5% vs 3%). While the primary focus of the study was on colorectal cancer, it demonstrated the clinical benefits of continuous VEGF inhibition in conjunction with a chemotherapy switch, thereby providing critical evidence for the “cross-line application” of anti-angiogenic therapy. This finding not only transformed the treatment paradigm for mCRC but also established a basis for investigating continuation treatment strategies in other solid tumors, such as breast cancer and non-small cell lung cancer. It suggests that anti-angiogenic therapy may necessitate a reevaluation of the conventional approach of discontinuing treatment upon disease progression.

In 2014, Rakesh K. Jain et al. (11) proposed a transformative approach to antiangiogenic therapy, illustrating that the inhibition of VEGF and its receptor (VEGFR) can temporarily enhance tumor perfusion and reduce hypoxia. His research demonstrated a correlation between improved tumor oxygenation and increased survival rates among glioblastoma patients, thereby challenging the traditional “tumor starvation” strategy. While his work primarily concentrated on cancer biology, it provides significant insights into the modulation of the microenvironment in chronic diseases. Jain’s identification of dose-dependent vascular normalization windows and the mechanisms underlying stromal decompression emphasize the importance of dynamic regulation within the microenvironment. Moreover, his integration of perfusion biomarkers and combination therapies with immunomodulators presents a framework for developing precision medicine strategies aimed at targeting pathological angiogenesis across diverse disease contexts. In the same year, the Falcone team (35) conducted the TRIBE trial, a Phase III randomized controlled study involving 508 previously untreated patients with mCRC. This study compared the efficacy and safety of two treatment regimens: FOLFOXIRI (fluorouracil, oxaliplatin, and irinotecan) with bevacizumab, versus FOLFIRI (fluorouracil and irinotecan) also with bevacizumab. The results showed that the median progression-free survival (PFS) for the three-drug combination was significantly longer at 12.1 months, compared to 9.7 months in the control group (HR = 0.75, p = 0.003). The objective response rate also improved by 12%, with 65% in the FOLFOXIRI group versus 53% in the control group (p = 0.006). Although overall survival did not reach statistical significance, there was a trend toward improvement, with 31.0 months for the three-drug group compared to 25.8 months for the control (p = 0.054). However, the three-drug regimen was associated with significantly higher rates of grade 3-4 neurotoxicity (5.2% vs. 0%), diarrhea (18.8% vs. 10.6%), and neutropenia (50% vs. 20.5%). This study confirmed the potential benefits of intensified chemotherapy combined with anti-angiogenic therapy as a first-line treatment for mCRC while highlighting the need to balance efficacy with toxicity risks. These findings provide a crucial evidence-based foundation for future treatment regimen selection and maintenance strategies.

In 2015, Cremolini et al. (36) conducted the Phase 3 TRIBE study to compare the efficacy of FOLFOXIRI in conjunction with bevacizumab versus FOLFIRI with bevacizumab as first-line therapies for metastatic colorectal cancer. Their updated analysis revealed that the FOLFOXIRI regimen significantly enhanced median overall survival (29.8 months compared to 25.8 months; HR 0.80, p=0.03) and progression-free survival (12.3 months versus 9.7 months; HR 0.77, p=0.006). Molecular subgroup analysis indicated that BRAF mutations were correlated with a poorer prognosis (median overall survival of 13.4 months); however, treatment efficacy remained consistent across RAS/BRAF subgroups (pinteraction=0.52). Although the study primarily focused on strategies for chemotherapy intensification, it also underscores the critical role of comprehensive molecular profiling in optimizing treatment regimens. The findings endorse the use of FOLFOXIRI/bevacizumab as a viable first-line therapeutic option irrespective of molecular status, thereby highlighting the necessity for tailored approaches in populations harboring BRAF mutations.

### 4.3 Emerging research trends and hotspots

The keyword analysis of AAT for CEC reveals a dynamic research landscape characterized by both established and emerging areas of focus. Traditional concepts, such as “vascular endothelial growth factor,” remain essential; however, new research directions have emerged in the past three to five years.

Biomarker-guided treatment optimization has become a pivotal aspect of colorectal cancer research. Casadei-Gardini A et al. (37) demonstrated that the colon inflammatory index (CII) can predict clinical outcomes for patients undergoing first-line chemotherapy with or without bevacizumab, establishing it as an independent prognostic factor. Tokunaga R et al. (38) investigated single nucleotide polymorphisms (SNPs) in the adenosine pathway and found that variants such as CD39 RS11188513 could influence clinical outcomes in patients treated with FOLFIRI plus bevacizumab. These findings enhance patient stratification and the tailoring of treatment strategies.

The exploration of combination therapies aims to improve treatment efficacy. Cortellini A et al. (39) reported on the FIRB/FOX regimen, which integrates 5-fluorouracil, bevacizumab, and alternating weekly doses of irinotecan and oxaliplatin. In a real-world setting, this regimen achieved a 75.9% objective response rate over three months and a median progression-free survival of 14.4 months, indicating its potential as an effective treatment option.

Research into treatment strategies for specific populations, such as elderly patients, is also increasing. Carrato A et al. (40) conducted a phase II trial with frail elderly patients suffering from advanced colorectal cancer, using regorafenib as first-line treatment. Although the pre-specified six-month progression-free survival rate was not achieved, the disease control rate and overall survival results were encouraging, suggesting that further investigation is warranted. Ongoing evaluations of the safety of existing drugs and the discovery of new targets are critical. Yamaguchi K et al. (41) conducted a large-scale study involving Japanese patients with metastatic colorectal cancer treated with regorafenib, identifying common adverse drug reactions such as hand-foot skin reaction, liver injury, and hypertension. Concurrently, Deng Fl et al. (42) identified DKK2 as a potential anti-angiogenesis target, stimulating angiogenesis through a VEGF-independent pathway and opening new avenues for treatment.

The search for more precise predictive biomarkers for treatment response continues. Studies on VEGFA splice variants and microRNAs have highlighted their potential to predict treatment outcomes (43). For instance, VEGFA145B was identified as a negative predictor of progression-free survival in right-sided tumors, while overexpression of miR-143-3P was associated with improved outcomes. Determining optimal treatment sequences is another critical area of research. A study comparing FOLFIRI-aflibercept and FOLFIRI-bevacizumab as second-line treatments for RAS-mutated patients found both regimens to be equally effective; however, FOLFIRI-aflibercept presented a numerically lower risk of death during the six-month induction phase. The BEVAMAINT trial is currently comparing maintenance therapies to identify the superior option for time-to-treatment failure (44). The interaction between the immune system and angiogenesis is also being explored. Studies indicate that neutrophil infiltration can counteract anti-VEGF therapy (45), while the combination of immune checkpoint inhibitors and anti-angiogenic agents, such as regorafenib and nivolumab (46), shows promise in treating advanced colorectal cancer.

### 4.4 Emerging therapies and future directions

The field is witnessing the emergence of novel AAT that focus on alternative pathways involved in VEGF-independent angiogenesis, such as DKK2 and TIE2. Preclinical investigations have identified DKK2 as a promoter of angiogenesis via VEGF-independent mechanisms, highlighting its potential as a therapeutic target (42). Similarly, TIE2 signaling has been recognized as a critical regulator of tumor angiogenesis, with inhibitors demonstrating promise when utilized in conjunction with standard therapeutic regimens (29). Furthermore, the integration of liquid biopsy techniques, including ctDNA, is revolutionizing the monitoring of treatment responses and the identification of resistance mechanisms. For instance, the CIRCULATE-PRO study employed ctDNA to pinpoint predictive biomarkers for response to aflibercept, facilitating more precise patient stratification (20). Advancements in nanotechnology-based drug delivery systems are also progressing, particularly with the development of pH-sensitive carriers aimed at enhancing the targeted release of VEGFR inhibitors within the tumor microenvironment, thereby potentially reducing systemic toxicity (22). These innovations underscore the shift towards personalized precision medicine in the treatment of CRC.

### 4.5 Clinical relevance and future research

While bibliometric analysis offers a quantitative overview of research trends, it is imperative to augment this approach with qualitative assessments of clinical impact. For example, the TRIBE study demonstrated that the combination of FOLFOXIRI with bevacizumab significantly improved survival compared to FOLFIRI with bevacizumab in specific patient subsets, thereby underscoring the importance of intensified chemotherapy (35). Such findings not only inform clinical practice but also guide the design of future clinical trials.

In conclusion, this bibliometric analysis provides insight into the dynamic landscape of AAT research in CRC, encompassing both fundamental mechanistic studies and innovative clinical applications. By addressing limitations and incorporating perspectives from gray literature and qualitative analyses, this study offers a comprehensive viewpoint that can assist researchers and clinicians in navigating the complexities associated with CRC treatment.

### 4.6 Limitations

This bibliometric analysis offers a comprehensive overview of the research landscape pertaining to AAT for CRC. However, it is important to acknowledge several limitations.

First, the study relied exclusively on the Web of Science Core Collection for data retrieval. While this database is esteemed for its coverage of high-impact, peer-reviewed journals, it may not encompass all pertinent literature, particularly from non-English sources or other databases such as PubMed and Scopus. Future research should consider integrating multiple databases to ensure a more comprehensive representation of the field. This approach could uncover additional studies, especially from non-English-speaking countries, and provide insights into region-specific clinical practices and emerging research trends.

Second, the exclusion of gray literature, including conference abstracts and clinical guidelines, represents another limitation. Gray literature often contains preliminary findings from early-phase clinical trials, interim results, or consensus recommendations that can offer valuable insights into evolving clinical practices and therapeutic strategies. For instance, abstracts from major oncology conferences such as ASCO and ESMO have highlighted the potential of novel AAT combinations with immunotherapy in CRC, suggesting promising directions for future research. Although these sources were omitted from our bibliometric analysis due to their non-peer-reviewed status, they are critical for understanding the current state of the field and potential future developments.

Third, bibliometric analysis primarily emphasizes quantitative metrics, such as publication counts and citation frequencies, which may not fully capture the qualitative impact or clinical relevance of the research. To address this limitation, we have supplemented our findings with qualitative discussions of key studies and their contributions to the field, aiming to provide a more nuanced understanding of the clinical implications of the research.

Finally, the study did not explore the heterogeneity among different AAT agents, such as bevacizumab, regorafenib, and aflibercept, which possess distinct mechanisms of action and clinical profiles. While our analysis identified general trends in AAT research, a more detailed examination of agent-specific developments could yield additional insights into their comparative effectiveness and roles in combination therapies. Despite these limitations, this study offers a robust overview of the AAT research landscape in CRC, highlighting key trends, collaboration networks, and emerging areas of focus that can inform future research and clinical practice.

## 5 Conclusion

This bibliometric analysis of 976 publications (1996–2024) reveals trends in antiangiogenic therapy (AAT) for colorectal cancer (CRC):

### 5.1 Research growth and impact

The field has grown at 13.65% annually, with China leading in publications (20.50%) but limited collaboration. Canada, Germany, and the UK show stronger networks. Research has evolved from VEGF studies to precision strategies targeting tumor microenvironments.

### 5.2 Mechanistic and clinical advances

VEGF/VEGFR Dynamics: Splice variants and changes inform biomarker strategies. Vascular Normalization: Optimal chemotherapy windows are identified. Resistance Mechanisms: CAF-derived IL-8 activation prompts targeted trials like Galunisertib. Clinical Innovations: Regorafenib’s dual inhibition and ctDNA-guided predictions advance precision therapy.

### 5.3 Emerging frontiers

Biomarker Integration: CII, VEGFA variants, and microRNAs enhance stratification. Combination Strategies: Immuno-angiogenic combinations and novel delivery systems improve efficacy. Population-Specific Protocols: Tailored approaches for specific patient groups are crucial.

### 5.4 Challenges and future directions

Further investigation into resistance mechanisms and AAT’s real-world applicability is needed. Future studies should address database biases and expand datasets for better interdisciplinary models.
